# *SIGLEC1* as a novel prognostic factor is regulated by Yiqi Huayu Jiedu Decoction in colorectal cancer

**DOI:** 10.7150/jca.99278

**Published:** 2025-01-01

**Authors:** Yejin Zhu, Xinping Wang, Qianyi Wang, Shanfan Shi, Jun He, Chao Jiang

**Affiliations:** 1 School of Medicine, Nanjing University of Chinese Medicine, Nanjing, 210023, China.; 2 Affiliated Hospital of Nanjing University of Chinese Medicine, Jiangsu Province Hospital of Chinese Medicine, Nanjing, 210029, China.; 3 School of Elderly Care Services and Management, Nanjing University of Chinese Medicine, Nanjing, 210023, China.

**Keywords:** *SIGLEC1*, Colorectal Cancer, Bioinformatics, Yiqi Huayu Jiedu Decoction, Traditional Chinese Medicine

## Abstract

We studied the prognostic value of *SIGLEC1* in colorectal cancer (CRC) using bioinformatics. *SIGLEC1* exhibited differential expression between the tumor and control samples, and improved survival was observed in patients with increased *SIGLEC1* expression. The univariate and multivariate analyses confirmed *SIGLEC1* as an independent prognostic factor for CRC, based on which a nomogram was constructed for predicting survival in patients with CRC. Additionally, higher *SIGLEC1* expression was correlated with increased immunological/stromal/ESTIMATE scores as well as immune cell infiltration and was strongly and positively associated with T helper cells and macrophages. Furthermore, significant positive correlations were observed between SIGLEC1 expression and inhibitory/coinhibitory immunological genes. Additionally, the TIDE results of patients with CRC showed that increased *SIGLEC1* expression was related to poorer immunotherapeutic responses. In clinical samples from patients with CRC, a decrease in *SIGLEC1* expression was noted compared to para-cancerous tissues and samples from patients who received Yiqi Huayu Jiedu Decoction (YHJD) treatment. The *in vivo* results indicated the superior efficacy of YHJD against CRC in inhibiting tumor metastasis. Our study demonstrates that SIGLEC1 serves as a prognostic factor for CRC, strongly linked to immune response, and can be modulated by YHJD, suggesting novel avenues for CRC treatment.

## Introduction

Colon cancer, also known as colorectal cancer (CRC), is a serious form of cancer typically arising at the junction of the rectum and sigmoid colon. This cancer frequently evolves from precancerous polyps in the colon 1. CRC ranks as the third most prevalent and the second most lethal cancer globally, with approximately 1.9 million people being diagnosed each year 2 Colon cancer development is attributed to complex related factors, among which the genetic factors should be attach importance to 3. Individuals suffering from colon cancer commonly endure gastrointestinal symptoms, including alterations in bowel habits, the presence of an abdominal mass, and abdominal pain, significantly impacting their everyday lives 4.

The accurate diagnosis of colon cancer and devising effective treatment options against it is a global concern among healthcare professionals. Current treatment strategies for CRC are chemotherapy, radiotherapy, and surgical resection, among which surgery is the most used approach. However, even after surgery, CRC exhibits a recurrence rate of 30% 5. Moreover, surgical resection may cause postoperative complications such as constipation or diarrhea owing to the removal of a part of the healthy colon 6. In summary, there is a lack of a comprehensive strategy for the early diagnosis and treatment of colon cancer patients, resulting in their prognosis remaining less than optimal. Therefore, future studies should be performed to elucidate the molecular mechanisms of colon cancer, screen new markers and therapeutic targets, and provide innovative methods for managing patients with colon cancer.

Sialic acid-binding immunoglobulin-like lectin 1 (*SIGLEC1*/CD169) is a receptor found on the surfaces of macrophages and dendritic cells, and its expression can be induced by interferons. It is a critical candidate gene for disease surveillance 7, 8. The SIGLEC family comprises transmembrane proteins that interact with sialic acid and contain C2-set immunoglobulin domains, thereby regulating the function and immune response of innate and adaptive immune cells 9, 10. Previous studies suggest that *SIGLEC1* contributes to the inflammatory immune response following a pathogen infection because it is elevated by inflammatory macrophages and affects T-cell activation and functions 11, 12. *SIGLEC1* is markedly related to inflammatory disorders, including asthma, pulmonary active tuberculosis, COVID-19, and chronic obstructive pulmonary disease 13, 14. Furthermore, *SIGLEC1* has been linked to conditions such as type 1 diabetes and systemic lupus erythematosus 15, 16. CD169, which is encoded by the *SIGLEC1* gene, is a protein found in a specific subset of macrophages located in secondary lymphoid organs. CD169+ macrophages elicit anti-tumor immune responses by capturing and presenting antigens derived from tumors, thereby inhibiting tumor growth and spread 17-19. Recent researches have shown *SIGLEC1*+ macrophages in CRC and hepatocellular carcinoma, suggesting their potential involvement in tumor progression 20, 21. CD169 may be a specific marker of inflammatory macrophages with pro-inflammatory properties. The pro-inflammatory cytokine, type 1 interferon, induces strong expression of CD169 in macrophages of CRC^22^. Despite these advancements, the therapeutic potential of targeting CD169 in CRC remains largely unexplored. Current research has focused primarily on elucidating the mechanisms underlying CD169's functional roles, with limited investigations into the development of direct CD169-targeted therapies for CRC prevention or treatment 23, 24. This gap highlights the need for further studies, particularly those aimed at translating our understanding of CD169's role in CRC into novel therapeutic strategies.

Traditional Chinese Medicine (TCM) that comprises multiple herbs with different active components, target sites, and pathway regulation activities can be a suitable treatment option against malignant diseases. Yiqi Huayu Jiedu Decoction (YHJD), a traditional polyherbal formulation, has been utilized in clinical practice since 1078 AD for addressing the pathogenesis of “spleen deficiency and stasis toxin” in CRC. 25. According to *Chinese Pharmacopoeia* (2020 Edition), each 200 mL of YHJD consists of 15 g of* Astragalus membranaceus* (Fisch.), 15 g of* Codonopsis pilosula* (Nannf.), 10 g of* Atractylodes macrocephala* (Koidz.), 15 g of* Poria Cocos* (Schw.) (Wolf.), 15 g of* Dioscorea opposita* (Thunb.), 5 g of* Prunus mume* (Siebold & Zucc.), 10 g of* Sparganium stoloniferum* (Graebn.), 10 g of* Curcuma phaeocaulis* (Valeton.), 10 g of* Agrimonia Pilosa* (Ledeb.), 30 g of* Patrinia villosa* (Juss.), and 30 g of* Glycyrrhiza uralensis* (Fisch.), and all these herb names are available at “World Flora Online” (www.worldfloraonline.org). Some recent studies have shown YHJD inhibitory effects on CRC, indicating its potential for preventing and treating gastrointestinal CRC with satisfactory efficacy 25-27. Zhou *et al.* indicates that YHJD can prohibit liver metastasis of CRC *in vivo*, especially through the PI3K-AKT signaling pathway and the nature killer (NK)-cell-led immune response 25. However, the active ingredients and underlying molecular mechanisms require further thorough investigation. 5-Fluorouracil (5-FU) is a crucial fluoropyrimidine antimetabolite drug that exerts complex antitumor effects by interfering with a key enzyme in the DNA synthesis process - thymidylate synthase, and by being mistakenly incorporated into RNA and DNA, leading to DNA strand breaks. 5-FU plays a significant role in the treatment of colorectal cancer and is also widely used in the treatment of various other cancers, including breast cancer, head and neck cancer, ovarian cancer, gastrointestinal tumors, and basal cell carcinoma 28. 5-Fluorouracil does show a limited overall response rate in monotherapy for colorectal cancer 29. This generally implies that, although they may produce therapeutic effects in some patients, the response rate of fluoropyrimidine drugs as a monotherapy is not sufficient to make it the preferred treatment option for all patients with colorectal cancer. Therefore, fluoropyrimidine drugs often play an adjuvant role in the treatment of colorectal cancer. They are frequently combined with other chemotherapy drugs, targeted therapeutic drugs, or immunotherapy drugs to improve treatment efficacy and patient survival rates. This combined treatment regimen can utilize the synergistic effects between different drugs to attack multiple growth and survival pathways of tumor cells, thereby more effectively inhibiting tumor growth and spread 30.

Thus, herein, our objective was to assess the prognostic significance and potential mechanism of *SIGLEC1* in CRC, and to explore the regulatory impact of YHJD on *SIGLEC1*, utilizing bioinformatics analyses for this purpose.

## Materials and methods

### Data source

To determine the expression pattern of SIGLEC1, we utilised The Cancer Genome Atlas (TCGA) database to select Primary Solid Tumor, Recurrent Solid Tumor, Solid Tissue Normal a total of 521 samples with expression profiles of 480 cancer tissue samples and 41 control samples, and excluded samples without survival information and duplicates. 430 cancer samples with Primary Solid Tumor and survival information were selected for survival analysis (**Table S1**). Meanwhile, the overall survival (OS) and clinic indicators were obtained from the TCGA-CRC. Subsequently, the CRC-related datasets, GSE39582 (annotation platform: GPL570) and GSE10972 (annotation platform: GPL6104), were collected from the Gene Expression Omnibus (GEO) database. The GSE39582 encompassed 566 colorectal cancer samples and 19 non-tumour colorectal mucosa samples. After excluding those lacking survival information and survival time data, a selection of 19 normal and 556 cancer samples was made to conduct a survival analysis (**Table S2**). Also, the GSE10972 included 24 primary tumors of CRC and 24 control samples. Of these, 430 CRC samples from TCGA-CRC (out of 463) and the 556 CRC samples from the GSE39582 dataset with available clinical information were analyzed for survival outcomes.

### Characterization of the involvement of SIGLEC1 in CRC

To investigate the role of *SIGLEC1* in CRC, the expression differences were compared between the CRC and normal controls in TCGA-CRC, GSE39582, and GSE10972 datasets by the Wilcoxon test (p < 0.05). Based on the median value of *SIGLEC1* expression, the CRC patients with OS in TCGA-CRC and GSE39582 datasets were divided separately into low- and high-SIGLEC1 expression groups. Subsequently, K-M analysis and the log-rank test were employed to compare the survival rates of patients across these two expression groups using the survminer (v. 0.4.9) R package (https://CRAN.R-project.org/package=survminer). Then, the expression levels of *SIGLEC1* in different clinical characteristics were compared by the Wilcoxon (two groups) and Kruskal-Wills tests (three groups or more), including age, race, sex, tumor stage, and p-TNM staging. Finally, we conducted univariate and multivariate Cox regression analyses to ascertain if *SIGLEC1* expression served as an independent prognostic factor using the survminer (v. 0.4.9) R package (HR ≠ 1, p < 0.05). This was followed by the construction of a prognostic nomogram using the rms (v. 6.5-0) R package (https://CRAN.R-project.org/package=rms). Moreover, the calibration curve was applied to evaluate the predictive ability of the nomogram model.

### Investigation of potential mechanisms of SIGLEC1 in CRC

As the aforementioned, we categorized the TCGA-CRC patients into low *SIGLEC1* expression and high expression groups based on the median value of *SIGLEC1* expression. Here, the differentially expressed genes (DEGs) were screened between the high- and low-*SIGLEC1* expression groups via the limma (v 3.56.2) R package 31. The log2 fold change (FC) values of each DEG were sorted from largest to smallest. Meanwhile, the and then Gene Ontology (GO) terms and Kyoto Encyclopedia of Genes and Genomes (KEGG) pathways gene sets were downloaded from the Molecular Signatures Database (MSigDB, https://www.gsea-msigdb.org/gsea/msigdb) as background gene sets. Afterward, the Gene Set Enrichment Analysis (GSEA) was performed by the clusterProfiler (v 4.10.0) R package (|normalized enrichment score (NES)| > 1, p < 0.05) 32. Additionally, we utilized the ESTIMATE algorithm to analyze and compare the immunological, stromal, and ESTIMATE scores between the low- and high-*SIGLEC1* expression groups by the Wilcoxon test (p < 0.05). Through the single-sample GSEA (ssGSEA) algorithm of GSVA (v. 1.46.0) R package 33, the fraction of 29 immune cells was calculated, and the Wilcoxon test was used to compare the fraction differences between the high- and low-SIGLEC1 expression groups (p < 0.05). Simultaneously, the association between *SIGLEC1* expression and immune-cell infiltration was studied using the Tumor IMmune Estimation Resource (TIMER, https://cistrome.shinyapps.io/timer/) database. Moreover, we employed immunologic signature gene sets as a reference to calculate the enrichment score (GSVA score) for each gene set in each sample of TCGA-CRC using the Gene Set Variation Analysis (GSVA) method. The correlation between *SIGLEC1* expression and the obtained GSVA scores was then determined through Pearson analysis (p < 0.05 and |cor| ≥ 0.4). Except for the above analysis, Spearman correlation analysis was performed to find *SIGLEC1*-correlated genes with p-value < 0.05 and |cor| ≥ 0.6, and the functions of the obtained correlated genes were analyzed with the help of clusterProfiler (v 4.10.0) R package.

### The effect of *SIGLEC1* on the immune therapy of patients with CRC

To analyze the effect of *SIGLEC1* on the immune therapy of patients with CRC, we identified the correlation between *SIGLEC1* expression and inhibitory/coinhibitory immune genes 34 by the Spearman method. Additionally, we compared the TIDE score and the number of immunotherapeutic responders between the low- and high-*SIGLEC1* expression groups and determined the correlation between *SIGLEC1* expression and the TIDE score via the Wilcoxon test (p < 0.05).

### Prediction of candidate drugs targeted to *SIGLEC1* in CRC

*SIGLEC1* was uploaded to the Comparative Toxicogenomics Database (CTD, https://ctdbase.org/), followed by candidate drug screening by upregulating or downregulating its expression. Next, the obtained drug-*SIGLEC1* pairs were imported into Cytoscape to construct a drug-gene network.

### Clinical samples

The tumor samples from all patients with CRC used in this study were provided by Jiangsu Provincial Hospital of Chinese Medicine, Affiliated Hospital of Nanjing University of Chinese Medicine (No.2022NL-110-02). From November 2021 to July 2022, surgically excised samples of tumor and para-cancerous tissue were gathered from 16 patients diagnosed with locally colorectal cancer (CRC) that were deemed resectable. The comprehensive clinicopathological profiles of all these patients are detailed in the supplementary in table S3. Treatment response was evaluated using Guidelines for Diagnosis and Treatment of Colorectal Cancer issued by the CSCO (Chinese Society of Clinical Oncology) and National Health Commission of the People's Republic of China 35. Tumor and para-cancerous tissue samples of the patients were immediately treated at 4°C. Following the addition of Trizol, the samples were preserved in an ultra-low-temperature refrigerator at -80°C for later RNA or protein extraction. Simultaneously, patient data including the patient's admission number, gender, age, CRC type, and treatment stage, were collected.

### Cell culture

Human colon cancer cells that could be used for the red fluorescence localization of nuclei (HCT-116-mkate2) were originally obtained from Cell Valley (Nanjing) Biotechnology Co., Ltd. The HCT-116-mkate2 cells were grown in a culture medium composed of 90% 1640 culture medium, 10% fetal bovine serum (FBS), and 1X antibiotics. They were maintained in an environment with 5% CO_2_ and O_2_ at a temperature of 37°C.

### HCT-116-mkate2 xenograft model

Specific pathogen-free male BALB/c nude mice (5-6 weeks old, 20 g, SLAC, Shanghai, China) were used in the experiment. HCT-116-mkate2 cells, numbering 7.0 × 10^6^, were suspended in 150 μL of Dulbecco's Modified Eagle Medium (DMEM) with 10% fetal bovine serum (FBS) and injected into the underarm regions of the hind limbs of the mice. The dimensions of the tumor were assessed every 2 days using a Vernier caliper.

At the end of 14 days, mice bearing tumors of similar sizes were randomly allocated to various groups, including the control group, 5-Fluorouracil (5-Flu), YHJD, and 5-Flu+YHJD groups, with 4 mice in each group. YHJD (3.4 g/mL and 0.2 mL/20 g) and 5-Flu (30 mg/kg) were administered once every 2 days. The YHJD formulation was prepared in accordance with the *Chinese Pharmacopoeia* (2020 Edition) and a previously reported method 27, and the dose was selected with reference to the comparison of the reported growth inhibitory effects of YHJD on CRC in the mice 25. During modeling and drug treatment, the mice in the control group were administered normal saline. After a week of treatment, the tumors of the tumor-bearing mice were surgically removed. The surgical incisions made on the mice were stitched up. Subsequently, YHJD was administered to them every two days over a period of one week, and all the mice were euthanized in the fifth week. The *in vivo* imaging system (IVIS) (Tanon ABL X5, Shanghai Tianneng Technology Co., Ltd.) was used to visualize the tumors on day 0 of modeling, the day before the surgery, and one week after the surgery.

### Western blotting

Proteins were isolated using lysis buffer, incubated in SDS buffer, and then separated through SDS-polyacrylamide gel electrophoresis. Following this, they were transferred to a PVDF membrane for further electrophoresis. *SIGLEC1*/CD169 antibody (ab312840) and β-actin antibody (ab8226) were purchased from Abcam (Cambridge, MA, USA). The immunoreactive protein bands were visualized by means of the GE 600 RGB.

### Quantitative Real-Time Polymerase Chain Reaction (RT-qPCR)

RNA extraction from liver tissue and cell samples was carried out using RNAiso Plus reagent (TaKaRa Biotechnology Co., Ltd, Dalian, China), adhering to the protocol provided by the manufacturer. Gene expression quantification was carried out using SYBR green PCR mastermix on a BioRad real-time PCR detection system (Veenendaal, The Netherlands). GAPDH levels served as the normalization standard.

### Immunohistochemical Staining

This method was executed in line with the procedures detailed in a previous report. The HRP-Polymer Conjugated anti-Mouse/Rabbit IgG complex was acquired from Maixin-Bio (Fuzhou, China), *SIGLEC1*/CD169 antibody(ab312840) and Ki67 antibody(ab15580) were purchased from Abcam (Cambridge, MA, USA). Analysis of the immunohistochemical sections was conducted using Mantra 1.01 software from Perkin Elmer (Waltham, MA, USA).

### Hematoxylin-eosin staining

Tumor tissues from colorectal cancer (CRC) patients were processed as follows: they were stained with hematoxylin (Shanghai Ruji Biotechnology Development Co., LTD) and eosin (Beijing Solarbio Science & Technology Co., Ltd.), fixed in 4% paraformaldehyde (Sinopharm Co., LTD.), and then dehydrated in a gradient of ethanol (Sinopharm Co., LTD.). Following this, they were embedded in paraffin wax (Sinopharm Co., LTD.) and sectioned into 4-µm thick slices. These sections were subjected to hematoxylin staining for 5 minutes, followed by a 5-minute rinse in running water, and then stained with eosin for 2 minutes. The process concluded with routine dehydration, vitrification, and neutral resin mounting (Cida Biotechnology Co., LTD, Guangzhou). The OLYMPUS imaging system (DP74, Olympus Corporation, Japan) was used to capture images.

### Statistical analysis

Statistical analyses were carried out using SPSS 19.0 software. The results were represented as the mean ± SD based on at least three independent experiments. In the experimental sections, to evaluate statistical differences between two groups, the unpaired Student's t-test was utilized. To make comparisons among multiple groups, we employed one-way ANOVA.

## Results

### Thirty-eight survival-related differentially expressed genes (DEGs) are identified in CRC

**Figure 1:**
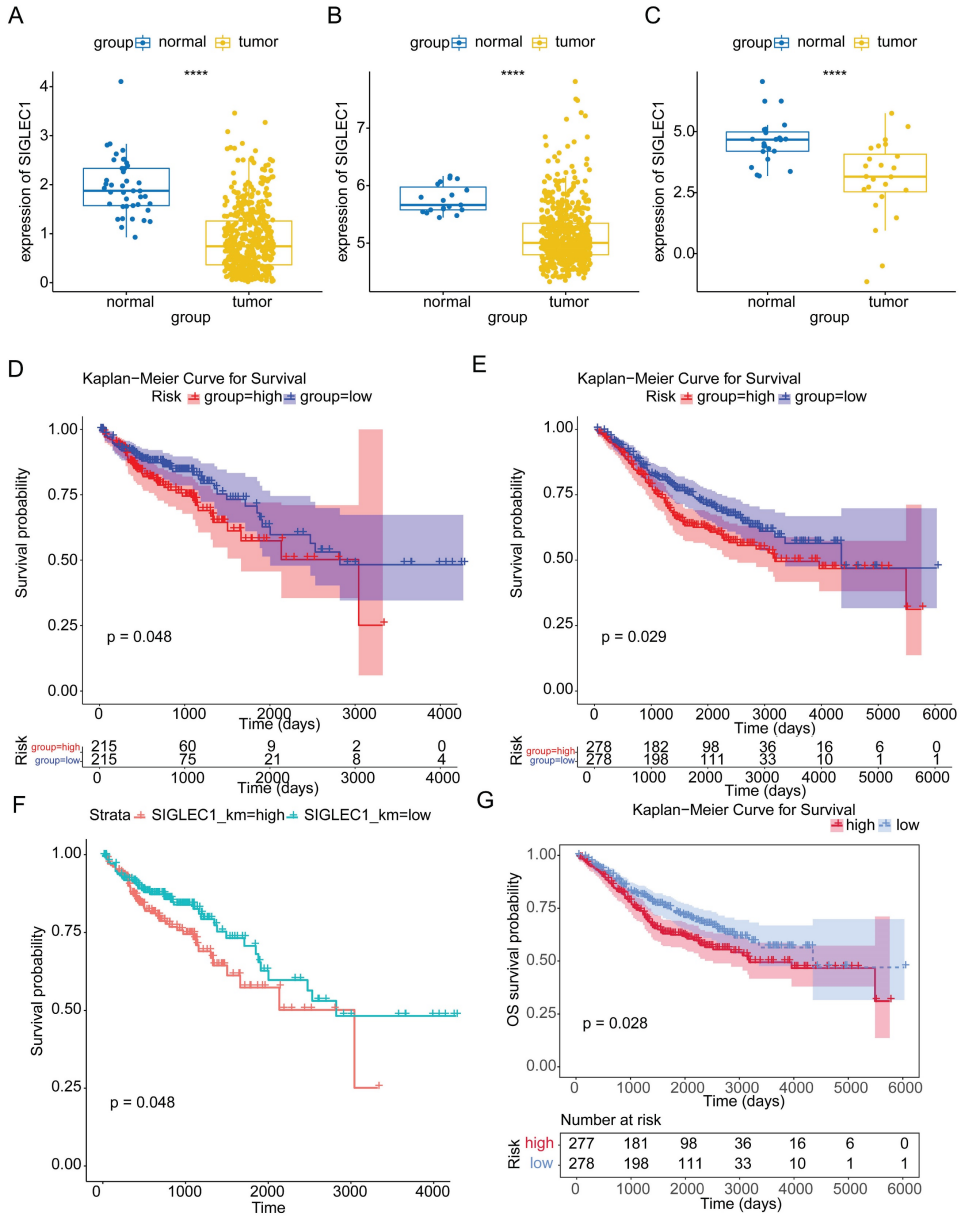
** Correlation between *SIGLEC1* expression and overall survival in colon cancer.** The expression of *SIGLEC1* in TCGA-CRC, **(A)** GSE10972, and** (B)** GSE39582. Kaplan-Meier survival analysis of **(D)** TCGA-CRC and **(E)** GSE39582. (**F-G**) Kaplan-Meier survival analysis of (F) TCGA-CRC and (G) GSE39582. “****” represents p < 0.0001.

*SIGLEC1* expression patterns were consistent among samples derived from TCGA, GSE39582, and GSE10972 cohorts. Gene expression in the tumor samples was markedly reduced compared to that in the control samples (p < 0.05) (**Figure 1A to 1C**). In patients with CRC, based on the median expression values of *SIGLEC1*, they were categorized into groups with low and high-*SIGLEC1* expression. Notably, CRC patients exhibiting lower SIGLEC1 expression demonstrated improved survival compared to those with higher* SIGLEC1* expression (p < 0.05), as observed in both TCGA and GSE39582 cohorts (**Figures 1D and 1E**). After eliminating the effects of gender and age (age is grouped according to the median value), although there was an individual in the GSE39582 data set with missing age information, it had no effect on the overall results (**Figure 1F and G**).

**Figure 2:**
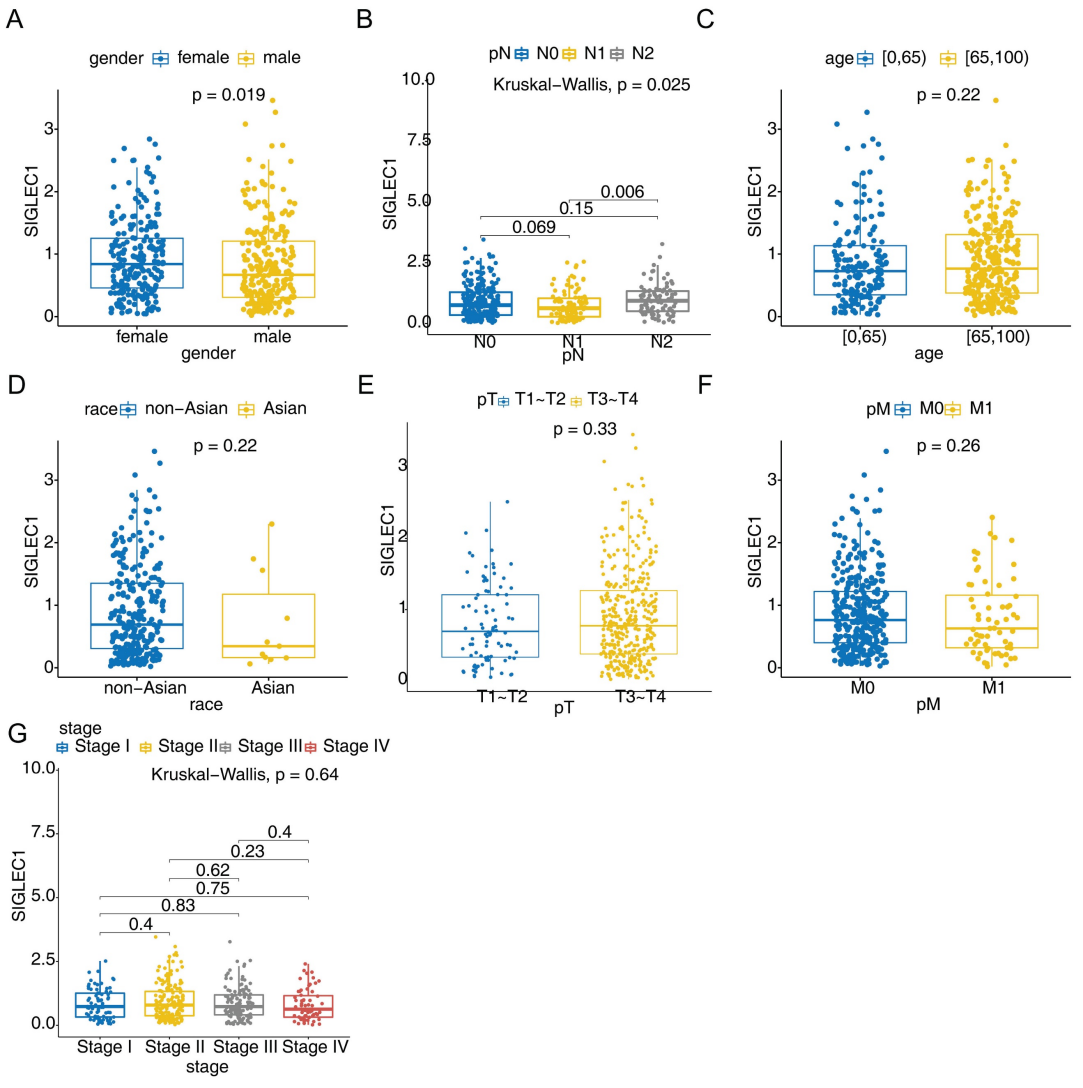
** The clinicopathological features of *SIGLEC1*.** These included **(A)** gender, **(B)** N stage, **(C)** age,** (D)** race, **(E)** T staging, **(F)** M staging, and **(G)** tumor stage.

Additionally, the association between *SIGLEC1* mRNA expression and the clinical characteristics of colon cancer patients in the TCGA cohort was examined. Among them, the *SIGLEC1* expression of female CRC patients was higher than that of male (p < 0.05) (**Figure 2A**), and there was a significant difference between the N1 and N2 stage (p < 0.05) (**Figure 2B**). However, no such correlation was observed for age (**Figure 2C**), race (**Figure 2D**), T staging (**Figure 2E**), M staging (**Figure 2F**), and tumor stage (**Figure 2G**). These results indicated that *SIGLEC1* expression was related to CRC clinicopathological features.

### SIGLEC1 was an independent prognostic factor for CRC

**Figure 3:**
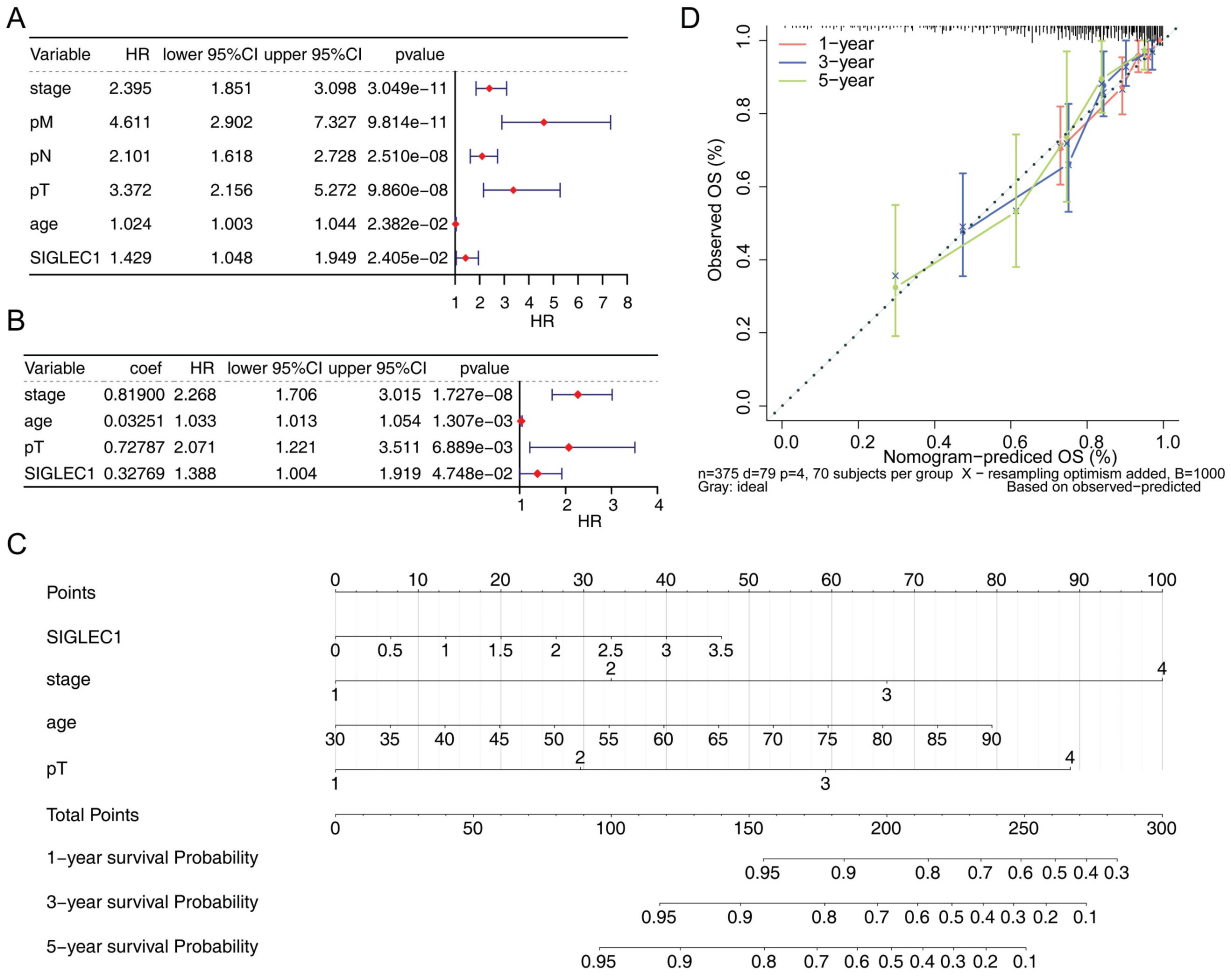
** Independent prognostic analysis of CRC.** The forest plot of** (A)** univariate and** (B)** multivariate cox analysis of the association between *SIGLEC1* expression and the overall survival in CRC patients.** (C)** Nomogram and** (D)** calibration curve of an independent prognostic model.

To investigate the independent prognostic factors affecting the prognosis of CRC, the univariate and multivariate Cox regression analyses were performed. Firstly, the outcomes from the univariate Cox regression analysis indicated that *SIGLEC1* expression, tumor stage, T/N/M stage, and age were linked to the survival rate of patients with CRC (**Figure 3A**). Moreover, multivariate Cox regression analysis uncovered a notable association between *SIGLEC1* expression and the survival rate of patients with CRC (**Figure 3B**). What's more, the age and pT were also independent prognostic factors for CRC. This suggested that *SIGLEC1* served as an independent prognostic factor for CRC. In addition, we created a nomogram that incorporated independent prognostic factors such as *SIGLEC1*, tumor stage, age, and T stage, to forecast the 1-year, 3-year, and 5-year survival rates of CRC patients (**Figure 3C**). The calibration curves indicated a close alignment between the predicted and observed survival rates (**Figure 3D**), underscoring the nomogram's clinical utility in predicting CRC survival.

### SIGLEC1 had a close relationship with the tumor immune microenvironment

**Figure 4:**
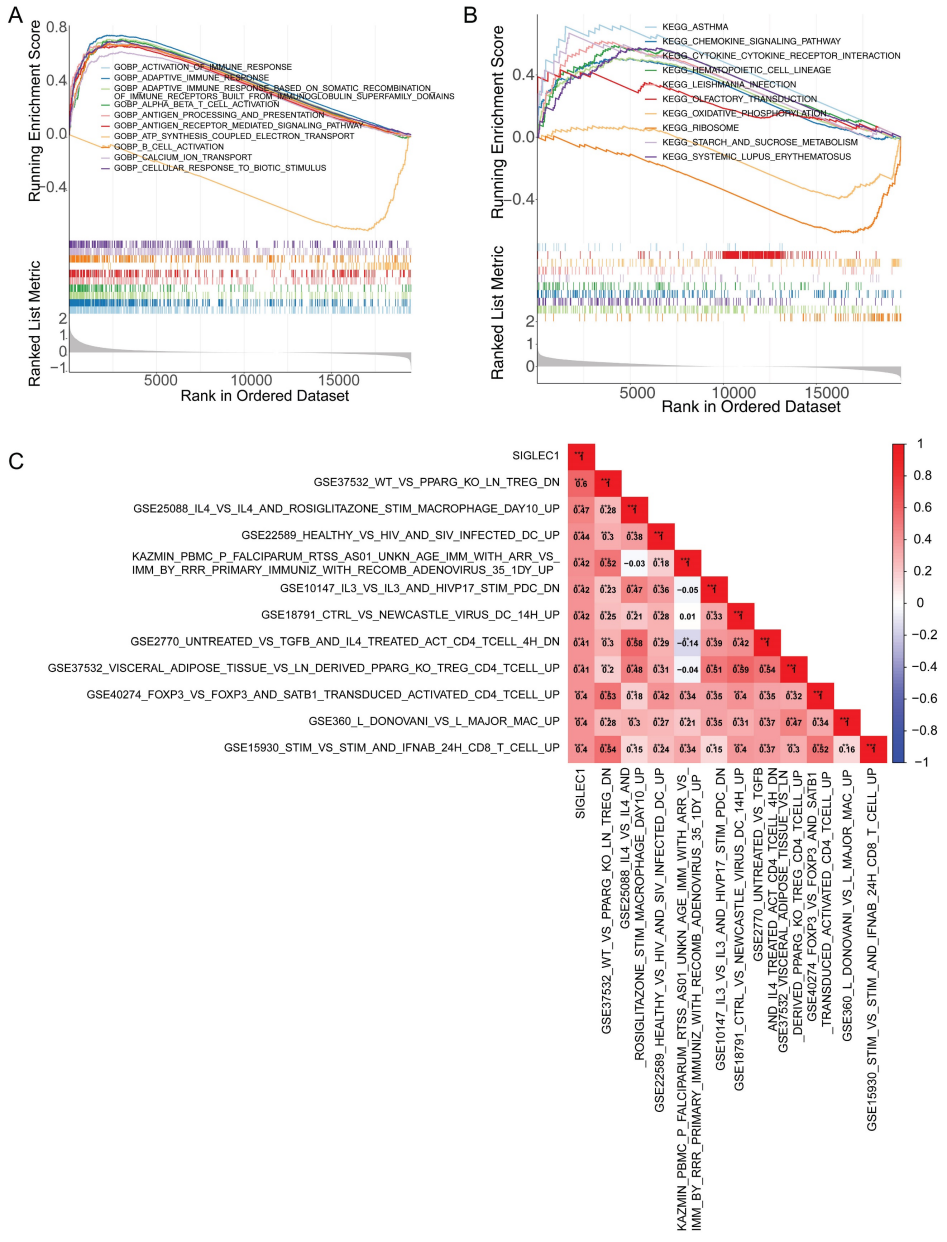
** Enrichment and Pearson's correlational analysis of** *SIGLEC1***. (A)** Enrichment results of *SIGLEC1* in GO.** (B)** Enrichment results of *SIGLEC1* in KEGG.** (C)** Pearson correlation of *SIGLEC1* with immune-related pathways.

The GSEA demonstrated that immune-response-related GO terms, like “activation of immune response,” “adaptive immune response,” “antigen processing,” and “antigen expression” (**Figure 4A**) and KEGG pathways, such as cytokine signaling and cytokine-cytokine receptor interaction (**Figure 4B**) were notably enriched in the low- and high-*SIGLEC1* expression groups, indicating a strong correlation between *SIGLEC1* and immunity. Additionally, the GSVA results indicated that *SIGLEC1* expression was positively associated with multiple immunologic signatures such as GSE37532_WT_VS_PPARG_KO_LN_TREG_DN, GSE25088_IL4_VS_IL4_AND_ROSIGLITAZONE_STIM_MACROPHAGE_DAY10_UP, GSE22589_HEALTHY_VS_HIV_AND_SIV_INFECTED_DC_UP, etc. (**Figure 4C**), which confirmed the close relationship between *SIGLEC1* and immune responses.

**Figure 5:**
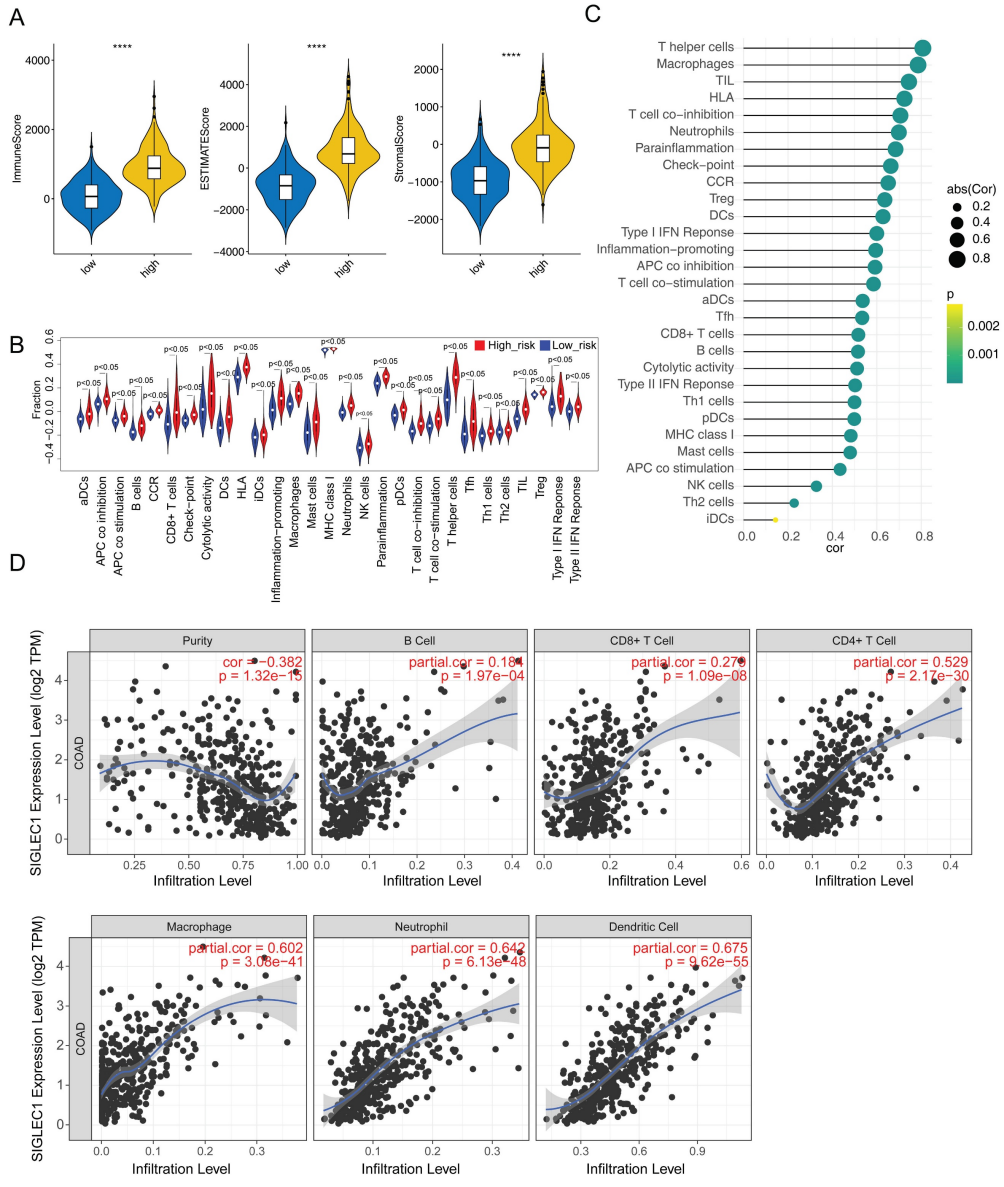
** Analysis of ESTIMATE and ssGSEA. (A)** The ESTIMATE algorithm was employed to assess the immune microenvironment in both the high and low expression groups. **(B)** The infiltration of immune cells in both the high and low expression groups was analyzed. **(C)** Relevance between *SIGLEC1* and immune cells.** (D)** Association between *SIGLEC1* expression and immune cells.

Additionally, we observed significantly increased immunological/stromal/ESTIMATE scores (**Figure 5A**) and immune cell and functional enrichment scores as well as enhanced pathways in the high *SIGLEC1* expression group (**Figure 5B**). Moreover, *SIGLEC1* expression was notably and positively associated with the 29 immune cells, functions, and pathways (**Table S4 and Figure 5C**). The TIMER results showed significant correlations between *SIGLEC1* and immune-cell infiltration (**Figure 5D**).

**Figure 6:**
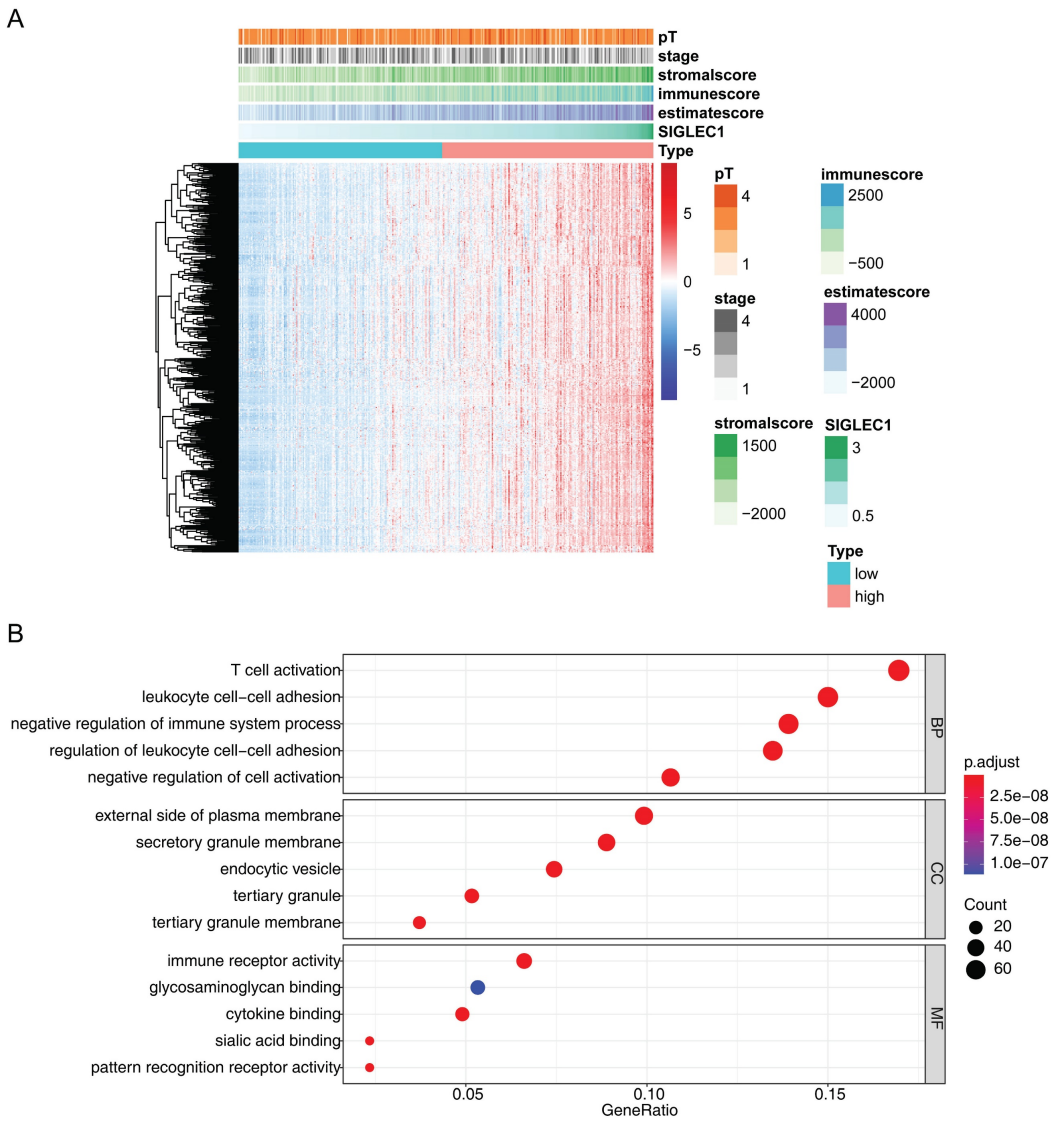
** Expression analysis of genes significantly strongly associated with and *SIGLEC1*. (A)** Heat map and **(B)** enrichment analysis.

Additionally, 493 *SIGLEC1* correlated genes (p < 0.05 and |cor| ≥ 0.6) were identified (**Figure 6A**), and their functions were notably enriched in immune-response-related GO terms, including “T-cell activation,” “leukocyte cell-cell adhesion,” and “immune receptor activity” (**Figure 6B**). Overall, these outcomes indicated that *SIGLEC1* played a crucial part in the CRC tumor immune microenvironment.

### SIGLEC1 affected immunotherapy of patients with CRC

Considering the above results, we investigated the role of *SIGLEC1* in the immunotherapy of patients with CRC and found a positive correlation between *SIGLEC1* and LAG3, CD38, PDCD1, KLRD1, TNFRSF4, FCRL4, CD200, and LGALS9 (**Figure S2A**), indicating that the patients with low *SIGLEC1* expression might exhibit weaker anti-tumor immunity. The TIDE method predicts the reactivity of immune checkpoint inhibitors; thus, it can be used to evaluate disparities in immunotherapy sensitivity between high- and low-expression groups 34. The present TIDE results showed that patients with high *SIGLEC1* expression exhibited higher TIDE scores and were more sensitive to immunotherapy (**Figure S2B**). Furthermore, *SIGLEC1* expression was positively correlated with the TIDE score (**Figure S2B**). Figure S2B showed a significant disparity in the number of responses between the groups with high and low expression. Next, we predicted 45 compounds that might regulate *SIGLEC1* expression and constructed a drug-*SIGLEC1* network (**Figure S2C**). Among the compounds predicted to upregulate the gene, bisphenol F (C000611646), estradiol (D004958), and genistein (D019833) were common active monomers in Chinese herbs. Moreover, the first-line antineoplastic drug, fluorouracil (D005472), was predicted to trigger *SIGLEC1* downregulation, which was likely to further weaken the immune response. Thus, these findings have implications for anti-CRC therapy.

### YHJD inhibited the development and metastasis of CRC via upregulated expression of SIGLEC1

**Figure 7:**
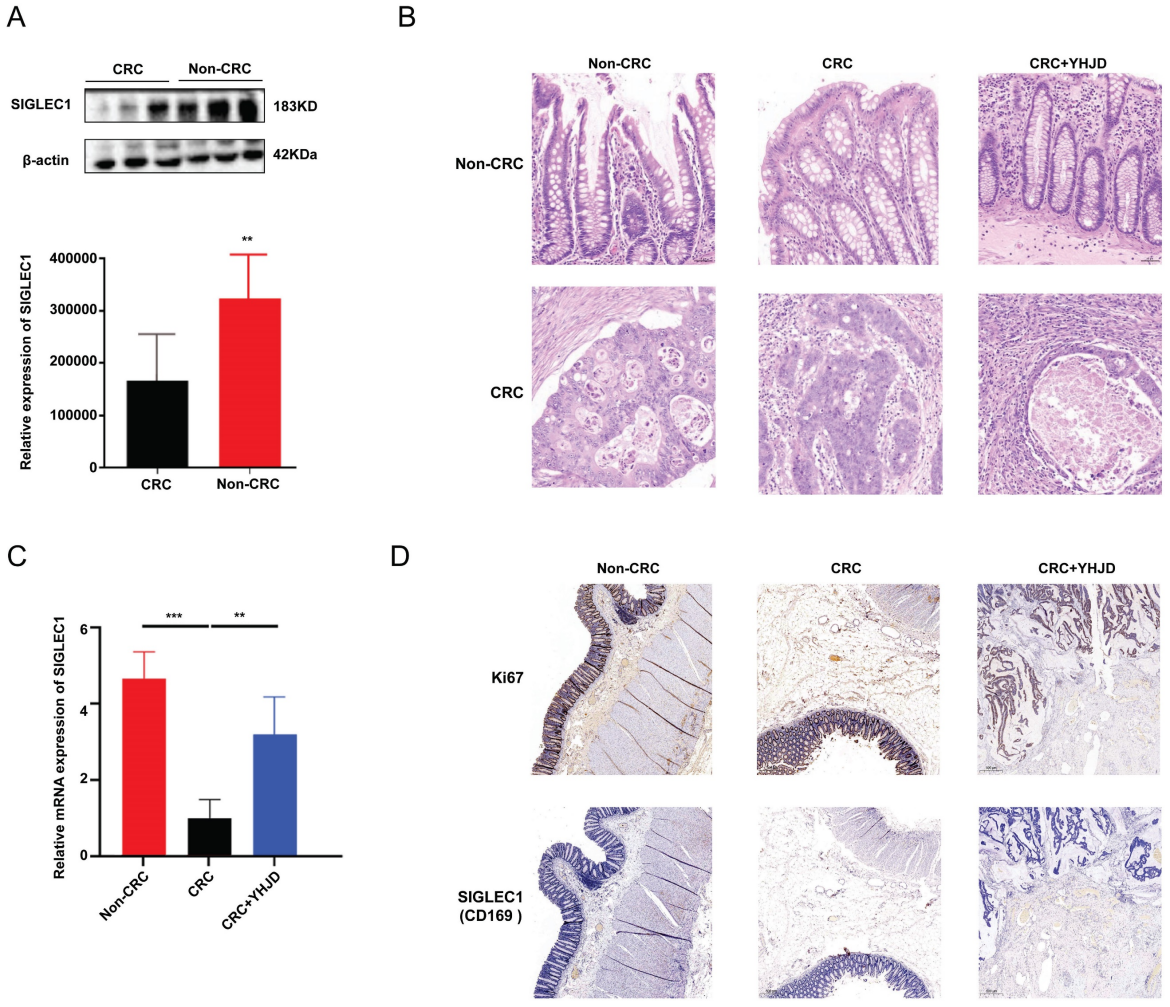
**In clinical terms, the *SIGLEC1* expression in CRC and the effect of YHJD. (A)** The protein expression of *SIGLEC1* was assayed by Western blotting, respectively. **(B)** Exploration of the infiltration of CRC using HE experiments. **(C)** The mRNA expression of *SIGLEC1* were assayed by RT-PCR, respectively. **(D)** Proteins expressions in the tumor were assessed by immunohistochemistry. Scale bar = 100 µm. (^**^p < 0.01, ^***^p < 0.001, n > 4).

We surgically collected CRC tumors and paracancerous tissues from 16 patients. The Western blot analysis results demonstrated that *SIGLEC1*/CD169 expression was lower in the CRC samples than in the non-CRC samples (**Figure 7A**, **B**). The PCR results showed that YHJD increased *SIGLEC1* mRNA levels in clinical samples obtained from the patients (**Figure 7C**). Relative to the non-YJHD treatment, YJHD treatment increased the expression of SIGLEC1/CD169, aligning with the findings from the database analysis (**Figure 7D**).

**Figure 8:**
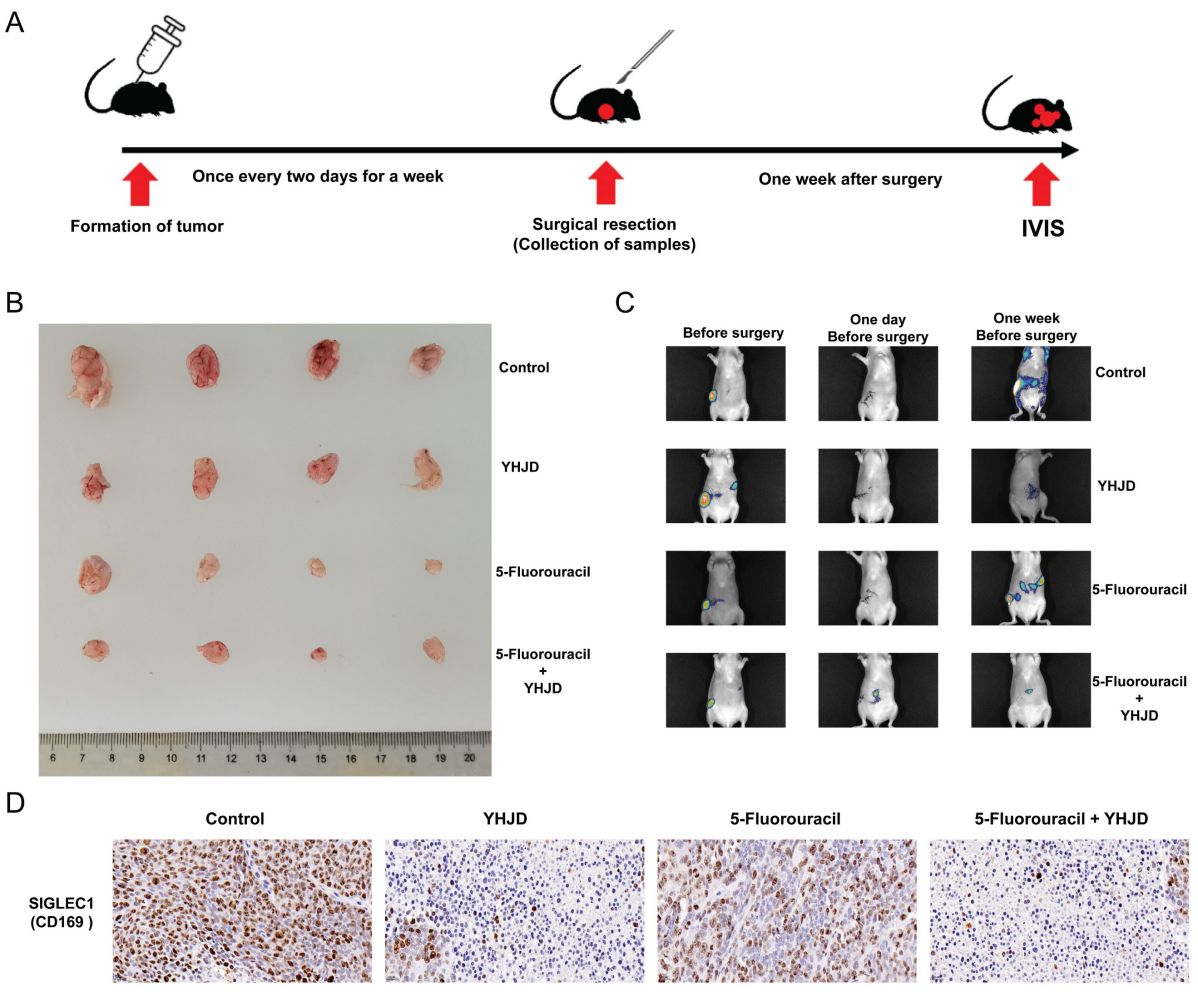
***In vivo*, the anticancer effects of YHJD on CRC. (A)** Experimental procedures for a CRC xenograft model. **(B)** Images of separated tumors from the control, YHJD only, 5-Flu only, and 5-Flu with the YHJD groups. **(C)** Fluorescence imaging was used to detect tumor growth and metastasis before and after the surgery.** (D)** Immunohistochemical methods were employed to detect the expression of proteins in tumors. (Scale bar = 20 µm. ^**^p < 0.01, ^***^p < 0.001, n = 4).

We performed an ectopic xenograft tumor assay to investigate the *in vivo* anticancer and metastasis inhibitory effects of YHJD. YHJD was administered every two days constantly after tumor formation in the inoculated cells (**Figure 8A**). A week following YHJD treatment, the tumor at the inoculation site was surgically excised. One week after the surgery, the tumors were detected using the IVIS, and the results indicated that the combined treatment of YHJD and 5-Fluorouracil (5-Flu) substantially suppressed tumor growth (**Figure 8B**) and metastasis, in comparison to the control group (**Figure 8C**). Immunohistochemistry assays were conducted to delve deeper into the effects of YHJD on CRC* in vivo* (**Figure**. **8D**). When YHJD was administered alone, the expression of *SIGLEC1*/CD169 was significantly upregulated. However, when combined with 5-Flu, this upregulation was even more pronounced. In contrast, the use of 5-Flu alone only induced a modest and non-significant upregulation of *SIGLEC1*/CD169. Consequently, it is evident that 5-Flu acts as an effective adjuvant, enhancing the ability of YHJD to upregulate *SIGLEC1*/CD169 expression. These findings indicate that YHJD plays a key role in treating CRC by upregulating *SIGLEC1*/CD169 expression and inhibiting tumor metastasis.

## Discussion

Herein, we first investigated *SIGLEC1* differential expression in both colon tumors and normal tissues, and the results showed significantly downregulated *SIGLEC1* expression in the tumor tissues than that in the normal tissues. Simultaneously, patients with colon cancer with lower *SIGLEC1* expression exhibited lower survival rates. The univariate and multivariate analyses confirmed the strong correlation between *SIGLEC1* and the survival of patients with CRC. To further validate these findings, we selected 10 patients with CRC and collected adjacent cancer tissues as a control. The PCR and western blotting results showed significantly low *SIGLEC1* expression in the patients with CRC compared with controls, which aligned with the results of the database analysis (figure 7A).

Additionally, we identified a positive correlation between *SIGLEC1* and various immunological characteristics of CRC. We observed that upregulated *SIGLEC1* expression was related to increased immunological scores, indicating improved immune responses and prolonged OS rates in the patients. The study found that the high-density infiltration of CD169-positive (CD169+) macrophages in the sinusoids of regional lymph nodes (RLN) is closely related to the increase of cytotoxic CD8 T cells in tumor tissues. The high-density infiltration of CD169+ macrophages is significantly associated with favorable overall survival (OS) in patients with colorectal cancer (CRC) 22. The significant increase in the number of SIGLEC1-binding cells observed in CRC mouse models is directly related to the disease state, revealing mechanism of CRC-induced changes in the immune microenvironment. As a key molecule for immune regulation, the increased expression level of SIGLEC1 may indicate more complex immune escape mechanisms and worse prognosis. Therefore, SIGLEC1 is expected to become an important biomarker for prognosis assessment in CRC patients and serve as a potential therapeutic target 36. A previous study has shown that *SIGLEC1* is predominantly abundant in immune process signaling pathways in patients with CRC 37, which is consistent with the present findings. Interestingly, a lower expression of* SIGLEC1* was observed in CRC patients, while a high-*SIGLEC1* expression group had a better prognosis. Here, we hypothesize that *SIGLEC1* showed inhibitory effects in the transition of normal cells in the colorectal mucosa to early CRC tumors. In tumor cells of CRC, *SIGLEC1* can further prevent the progression of tumors by promoting the activity of immune cells. We found a significant positive correlation between SIGLEC1 expression and inhibitory/co-inhibitory immune genes, which further emphasizes the potential role of SIGLEC1 in CRC immune evasion and immunotherapy resistance. This discovery provides new insights into the mechanisms of CRC immune evasion and may guide the development of novel immunotherapies targeting these mechanisms. In particular, we found that the expression levels of male CRC were much lower, therefore, these individuals were more likely to develop early CRC. These findings were consistent with the consensus that males were more likely to develop CRC 38. These results suggest that *SIGLEC1* may be a viable candidate for cancer immunity research and a target for cancer immunotherapy.

Lately, there has been an increasing emphasis on the role of the tumor microenvironment in the emergence and progression of cancer, especially in colon cancer. Studies have revealed that immune-cell infiltration can serve as a promising prognostic and therapeutic biomarker 39-42. Figure 5 shows a strong positive correlation between *SIGLEC1* expression and T helper (Th) cells, macrophages, and tumor-infiltrating lymphocytes (TIL). Similarly, the presence of Th17 cells and interleukin (IL)-17 was probably associated with the progression of different malignancies such as CRC, lung cancer, breast cancer, ovarian cancer, and gastric cancer 43-46. A notable reduction in Th17 cells was detected in CRC patients, which aligned with the decreased expression of *SIGLEC1* 47. Additionally, colon cancer cells can stimulate IL-1β production from THP1 macrophages, activating the Wnt signaling pathway and promoting cancer cell growth 48. Moreover, the presence of TILs has been recognized as a prognostic indicator for survival in patients with CRC. indicating that increasing TIL density is associated with improved survival rates 49. Additionally, the DEGs were enriched in the differentiation and chemokine signaling pathway of Th17 cells, which play a vital role in colon cancer development and advancement by producing and releasing cytokines 50-52.

The role of *SIGLEC1* was notably enriched in GO terms related to immune response, such as “T-cell activation,” “leukocyte cell adhesion,” and “immune receptor activity” (Figure 6). T-cell activation encompasses both the stimulation and inhibition of checkpoint signals. Elevated T-cell counts lead to an increased number of T cells, including cytotoxic lymphocytes and Th1 cells, which are related to improved survival rates in patients with CRC 53. The levels of activated leukocyte adhesion molecules in CRC tissues are higher than those in the normal mucosa, which is an independent prognostic factor for CRC 54. Additionally, immune receptors can suppress tumor growth by decreasing colonic tumorigenesis and key tumor-promoting signals. Therefore, their activity is critical for carcinogenesis and progression 55.

Among the predicted upregulated molecules, we focused on bisphenol F, genistein, and estradiol (Figure S2C). Bisphenol F is present in many TCM plants 56. Estradiol and genistein are active monomers commonly found in legumes and other TCM herbs 57.

YHJD is a TCM formulation developed based on colon cancer etiology, “spleen deficiency and blood stasis.” It originates from the classic prescription “Gui Shao Liujun Decoction” in the Song Dynasty's “Taiping Imperial Prescription” (1078 AD). A prior study has provided confirmation that YHJD effectively inhibits epithelial-mesenchymal transition and stem-cell formation in CRC by modulating the hsa-miR-374a-3p/Wnt3/β-catenin signaling pathway 27. Furthermore, YHJD can inhibit liver metastasis of colorectal cancer (CRC) *in vivo*, and its therapeutic effect is closely related to the regulation of multiple targets and effector processes, especially the PI3K-AKT signaling pathway and the immune response dominated by natural killer (NK) cells 25. Figure 8 displays a strong correlation between YHJD and *SIGLEC1* in clinical and animal CRC samples, indicating that YHJD inhibits *SIGLEC1* expression and restricts tumor growth and metastasis. Therefore, this study provides a basis for further studies on the effects and underlying mechanisms of YHJD. Currently, most studies focus on the functions of SIGLEC1 itself and its interactions with other immune molecules, rather than the regulatory mechanism of YHJD on SIGLEC1. Our findings not only uncover the pivotal role of YHJD in immunomodulation but also open up novel avenues for further elucidating its antitumor mechanisms. We plan to utilize high-throughput sequencing, proteomics, and other technologies in future studies to further uncover the underlying molecular mechanisms. Through *in vitro* cell experiments and *in vivo* animal models, we will verify the effects of YHJD upregulating SIGLEC1 expression on immune cell function and how this effect promotes anti-tumor immune responses. Additionally, we can combine preclinical research and clinical trial data to evaluate the potential clinical application value of YHJD as an anti-tumor drug or immune modulator.

In investigating the correlation between SIGLEC1 and tumors, despite meticulous efforts to select representative and rigorously screened samples, tumor heterogeneity remains a complex and largely uncontrollable factor. This intratumoral heterogeneity can lead to varying SIGLEC1 expression levels across different regions or cells, potentially influencing the composition and function of the tumor microenvironment, thereby complicating and obscuring the relationship between SIGLEC1 and its microenvironment. Additionally, variations among samples from diverse patient populations, stemming from factors such as genetic backgrounds, environmental exposures, and treatment histories, can also contribute to differences in SIGLEC1 expression levels. To better mitigate the influence of tumor heterogeneity and sample source variations on results, future endeavors should integrate multiple technologies and methodologies (e.g., genomics, transcriptomics, proteomics) to comprehensively analyze tumor heterogeneity. Experimental validation and clinical data comparison will be employed to multidimensionally verify and assess analytical outcomes. While we strive to manage confounding factors, analytical methods inherently possess assumptions and limitations that may not fully eliminate confounding effects. Moreover, complex interactions among confounding factors may be difficult to fully capture and explain within current analytical frameworks. Consequently, more advanced statistical approaches like machine learning and causal inference are being considered to enhance the control of confounding factors and improve the accuracy of results. Larger-scale, multi-center studies will be conducted to gather broader and more comprehensive data, facilitating better control of confounding factors and validating the universality of research findings. Furthermore, more detailed subgroup analyses and exploration of potential biological mechanisms can be undertaken to deepen our understanding.

Herein, SIGLEC1 has been acknowledged as a standalone prognostic factor for CRC by multiple analyses including bioinformatics analysis, clinical sample verification, and corresponding animal experiments. Our study demonstrated that SIGLEC1 not only serves as an independent prognostic factor but also displays a strong correlation with immunological parameters and immune cell infiltration within the tumor microenvironment. Importantly, our findings extend beyond mere correlations by uncovering a potential role for SIGLEC1 in modulating immunotherapeutic responsiveness, an area that has been understudied in CRC. Furthermore, our investigation represents a pioneering effort in exploring the therapeutic potential of TCM, specifically YHJD, in modulating SIGLEC1 expression and thereby influencing CRC progression.

## Conclusion

Our study offers a fresh perspective on CRC prognosis and therapy, with implications that extend beyond SIGLEC1 itself to the broader field of cancer immunology and TCM-based anticancer therapies. Nevertheless, additional research is required to gain a comprehensive understanding of the impact of YHJD on the tumor microenvironment and immune response.

## Supplementary Material

Supplementary figure and table legends.

Supplementary tables.

## Abbreviations

BSA: Bovine serum albumin
COPD: Chronic obstructive pulmonary disease
CRC: Colorectal cancer
CTLs: Cytotoxic lymphocytes
DEGs: Differentially expressed genes
GEO: Gene Expression Omnibus
GO: Gene Ontology
GSEA: Gene set enrichment analysis
GSVA: Gene set variation analysis
IVIS: *In vivo* imaging system
KEGG: Kyoto Encyclopedia of Genes and Genomes
K-M: Kaplan-Meier
M stage: Metastasis stage
N stage: Node stage
OS: Overall survival
SIGLEC1: Sialic acid binding immunoglobulin-like lectin 1
ssGSEA: Single-sample gene set enrichment analysis
T stage: Tumor stage
TCGA: The Cancer Genome Atlas
TCM: Traditional Chinese Medicine
TIDE: Time-series Dense Encoder
YHJD: Yiqi Huayu Jiedu decoction
