# Supplementary material for: SIGLEC1 as a novel prognostic factor is regulated by Yiqi Huayu Jiedu Decoction in colorectal cancer
Source: J Cancer. 2025 Jan 1;16(1):351–67. doi: 10.7150/jca.99278 (PMC11660119; doi:10.7150/jca.99278)
Supplement: Supplementary file 1 — Supplementary figure and table legends. [file jcav16p0351s1.pdf]

SIGLEC1 as a novel prognostic factor is regulated by Yiqi Huayu Jiedu Decoction in colorectal cancer

Yejin Zhu[a,*], Xinping Wang[b], Qianyi Wang[a], Shanfan Shi[c], Jun He[a,*], Chao Jiang[b,*]

[a] School of Medicine, Nanjing University of Chinese Medicine, Nanjing, 210023, China

[b] Affiliated Hospital of Nanjing University of Chinese Medicine, Jiangsu Province Hospital of Chinese Medicine, Nanjing, 210029, China

[c] School of Elderly Care Services and Management, Nanjing University of Chinese Medicine, Nanjing, 210023, China

Content:

1. Figure S1: SIGLEC1 affects immunotherapy of CRC patients

2. Table S1: Detailed clinical parameters of the TCGA-COAD sample

3. Table S2: Detailed Clinical Parameters of the GSE39582 sample

4. Table S3: Clincial characteristics of patients with colorectal cancer

5. Table S4: A total of 29 immune cells, functions, and pathways significantly associated with SIGLEC1

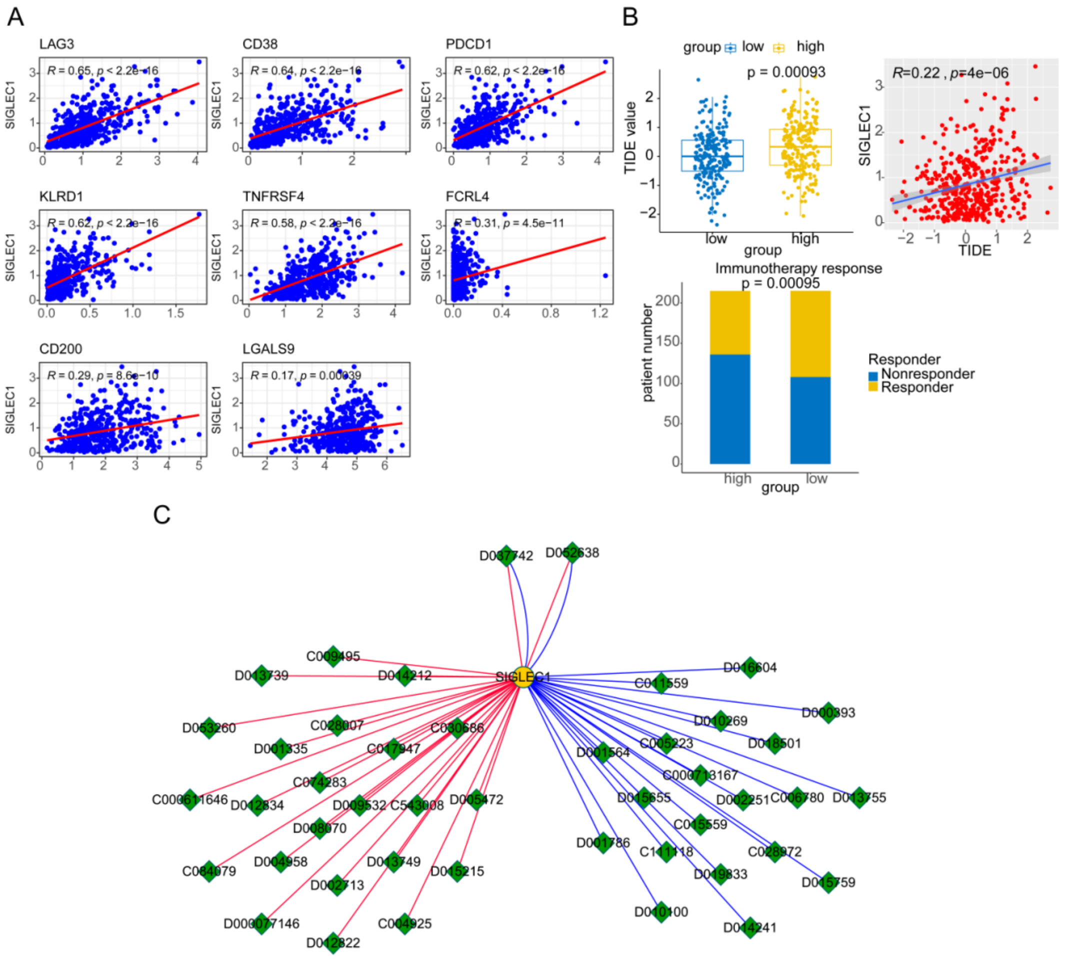

Figure S1. SIGLEC1 affects immunotherapy of CRC patients. (A) association of SIGLEC1 with inhibitory and co-inhibitory immune genes. **(B)** Analysis of TIDE score in high and low expression groups. **(C)** SIGLEC1 and drug network diagram.Yellow circles are genes, green diamonds are drugs, red lines represent effects of drugs on genes that increase expression, and blue lines represent effects of drugs on genes that decrease expression.
